# Preventing Zoonoses: Testing an Intervention to Change Attitudes and Behaviors toward More Protective Actions

**DOI:** 10.3390/ijerph20216987

**Published:** 2023-10-27

**Authors:** Marielle Stel, Nicole Banach

**Affiliations:** Department of Psychology of Conflict, Risk, and Safety, University of Twente, 7522 NJ Enschede, The Netherlands

**Keywords:** zoonoses, risk knowledge, health communication, attitudes, behavior change, protective behaviors, animals, public health, disease prevention

## Abstract

Zoonotic outbreaks are considered one of the most important threats to public health. Therefore, it is important to educate people on how to prevent zoonotic infections. The purpose of this research was to investigate an intervention aimed at changing people’s attitudes and behaviors toward more protective actions. In two studies (*N_Study_*_1_ = 402; *N_Study_*_2_ = 706), participants received an intervention based on previous literature in which knowledge about zoonoses, protective actions they could take, and a fear appeal were provided. In the control condition, no intervention was given. Subsequently, we measured participants’ risk knowledge, attitudes and behavioral intentions to reduce zoonotic risks, and fear. The results showed that the intervention heightened participants’ zoonotic knowledge and affected their attitudes and behavioral intentions (Studies 1 and 2) and a behavioral decision (Study 2) to reduce zoonotic risks. Moreover, our designed intervention proved more effective than the World Health Organization informative message on zoonoses (Study 2). In terms of theory, this is the first experimental demonstration that a general zoonotic risk communication message changed attitudes and behaviors toward more protective actions. In terms of policy, this research showed that a basic information message for the broader public has the potential to reduce zoonotic risks.

## 1. Introduction

Zoonotic outbreaks have become more frequent and carry more consequences [1,2]. Seventy-five percent of all emerging human infectious diseases originate from animals [3]. They are responsible for about one billion cases of illness in people and millions of deaths each year [4]. Therefore, it is important to educate people on which actions they can take to prevent future zoonotic outbreaks. The purpose of the current paper was to investigate an intervention aimed at heightening people’s zoonotic risk knowledge and changing their attitudes and behaviors toward more protective actions.

### 1.1. Zoonoses

Zoonoses are defined as diseases and infections caused by pathogens that are transmitted between animals and humans [5,6]. All types of pathogens can cause zoonoses, including viruses, bacteria, and parasites [5,7]. Possible hosts for these pathogens are a wide variety of animals, including pets, livestock, and wildlife [7]. These hosts can act as a disease reservoir (i.e., a natural long-term host) which results in a continuous source of human infections (e.g., [8,9]). Zoonoses can be transmitted via direct contact with infected animals or infected animal materials, via food, water, air, or via arthropods (e.g., mosquitos).

Zoonoses can be classified according to the ecosystem they circulate in [6]. The first classification is synanthropic zoonoses, which typically have an urban cycle. An example of a synanthropic zoonosis host is livestock kept domestically for the consumption of animal products such as meat, dairy, and eggs. Livestock animals can infect consumers of animal products and people who are on or near a farm or animal transport. Most zoonoses of recent concern have emerged from animal farming [10]. Zoonotic risks exist for all types of farming (e.g., [11]). In backyard and organic farming, animals can come into contact with other animals, leading to possible exposure to zoonotic pathogens carried by rats, birds, or other wild animals [11,12]. In intensive farming, these risks are decreased as the animals have no outdoor access. Despite biocontainment and biosecurity, however, zoonotic risks are not eradicated [13]. Even more so, the advantages of modern intensive farming do not outweigh the disadvantages, as intensive farming amplifies zoonotic risks (e.g., [11,14,15]). Zoonotic risks are amplified due to the high animal density at which they are kept [16]. Moreover, intensively farmed animals have a weaker immune system as a result of their genetic proximity (i.e., low genetic divergence) and the stressful conditions they live and are transported in, which increases zoonotic risks [17,18,19]. The emergence of zoonoses increases alongside enhanced demands for animals and their products [1,4,10]. Therefore, the solution lies in reducing the consumption of animals and their products (e.g., [11,15,20]).

Another example of synanthropic zoonosis hosts are pets, such as dogs and cats. Owners come into physical contact with their pets by stroking the pet, by being licked by their pet, and by sleeping together in one bed. These circumstances seem harmless but could lead to infections, such as cat scratch diseases, heartworms, intestinal diseases (e.g., toxoplasmosis, giardiasis), and vector-borne diseases transmitted by, for instance, fleas [21]. Regular visits to veterinarians for pet health checks and being educated about minimizing the transmission of zoonoses may help reduce the risks [21]. Furthermore, zoo animals are possible vectors for the transmission of zoonotic diseases [22,23]. In petting zoos, risky behavior includes hand-to-mouth contact after physical contact with animals and their environment. This is also a risk for caretakers and visitors to zoos with wild animals. So, reducing physical contact with animals and their environment and adequate hygiene and hand washing reduces zoonotic transmission [22,23].

The second classification is exoanthropic zoonoses with a sylvatic (feral and wild) cycle [6]. The infection source lies outside human habitats. For example, zoonotic risks exist when people hunt, butcher, and consume wild animals [2]. Hunters get into direct contact with wild animals and may be exposed to bodily fluids, bodily tissue, or feces, which spread pathogenic agents. Zoonoses also pose a risk due to the expansion of agricultural land for pasturage and growing food for farmed animals, as wild animals lose their habitats. This increases animal–human contact and, thus, zoonotic infections [11,24]. Finally, the consumption and transportation of wild as well as domestic animals could lead to zoonotic transmissions [18,25,26].

Thus, actions people could take to reduce zoonotic outbreaks are reducing the consumption of animals and their products, regular pet visits to vets, reducing physical contact with (wild) animals and their environments, and hygiene and hand washing [15,20,21,22,23].

### 1.2. Risk Knowledge, Attitudes and Behaviors toward Protective Behaviors

Zoonotic infections are considered one of the most important threats to public health [5]. Therefore, it is important that people are aware of the risks zoonoses pose so that they can choose to adjust their behavior to prevent future zoonotic outbreaks. In general, people’s risk perception with regard to various disasters and crises is low [27,28,29]. People make errors in risk evaluations as their estimations are influenced by subjective perceptions, intuitive judgments, feelings, and inferences made from limited information and media coverage (e.g., [30,31,32,33]). Regarding the risks of epidemics and pandemics, researchers have argued that the risks are generally underestimated [27,28,34]. Concerning zoonoses, several studies showed that people’s limited knowledge of zoonoses is responsible for their inaccurate risk perceptions [35,36,37,38,39]. For instance, the majority of dog owners do not know which parasites exist, which mechanisms cause their transmission, and which factors contribute to zoonotic infections [37]. Also, a study investigating people’s knowledge about Methicillin-Resistant Staphylococcus Aureus (MRSA, i.e., bacteria which are resistant to many antibiotics, causing infections) showed that participants had knowledge gaps regarding prevention, reservoir, spread, and origin of MRSA [39].

It is important to educate people to expand their knowledge and to increase their protective behaviors. Previous research showed that descriptions of diseases that emphasized specific zoonotic origins increased people’s risk perceptions and intentions to change their behaviors to avoid the diseases (e.g., [40], see also [41,42]. These information messages focused on specific zoonoses (for instance, Ebola) and provided information by describing the disease and the origin of the disease. In the present research, we focused on increasing risk knowledge, attitudes toward protective behaviors, and intentions to change these behaviors for zoonoses in general. In addition to providing people with information about zoonoses and their origin, it is important to educate them on which actions are necessary for prevention (e.g., [43,44]). Furthermore, risk perception can be increased by making use of a fear appeal.

A fear appeal is a communication message intended to elicit fear (e.g., [45]). Prominent theoretical models on people’s motivation to take protective actions make predictions about the impact of fear appeals. For instance, the Protection Motivation Theory (PMT) suggests that fear appeals initiate two processes: appraisals of threat and coping (e.g., [46]). Threat appraisals include the factors “perceived probability of the threat” and “severity of the threat”. Coping appraisals include the factors “perceived self-reliance or self-efficacy” and “effectiveness of the recommended behavior”. PMT suggests that people are motivated to protect themselves depending on these factors ([47,48]; for meta-analytic evidence, see [46,49]). The PMT factors mentioned are also the core factors that, according to the Extended Parallel Process Model (EPPM), determine whether a risk communication message is effective [50] (for a meta-analysis, see [45]).

In sum, previous research showed that providing people with information, educating them about the actions necessary for prevention, and eliciting fear by focusing on the threat (probability and severity of the threat) and on coping with the threat (self-efficacy and effectiveness of the recommended behavior) increased people’s intentions to take protective actions [40,41,43,44,51,52,53]. Therefore, in our intervention, we included these aspects. Note that our aim was to include these aspects, not to maximize these, as we aimed to provide a scientifically accurate information message. To our knowledge, we are the first to experimentally investigate the effect of a general zoonotic risk communication message on attitudes and behavioral intentions to take more protective actions.

### 1.3. The Present Research

**Figure 1:**
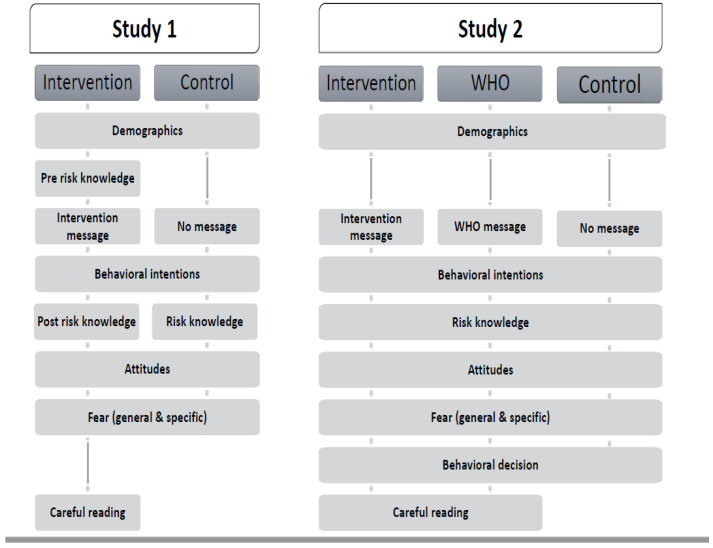
Overview of the sequence of events for both studies.

The present research consisted of two studies in which participants either received an intervention or no intervention. In the intervention condition, we provided people with knowledge on zoonoses (i.e., key facts and consequences), which actions they could take, and a fear appeal. The message included information on the probability and severity of the threat, self-efficacy, and effectiveness of the recommended behavior. In the control condition, participants did not receive any information regarding knowledge, action, or fear. So, the intervention and control conditions differed on several factors that were expected to increase risk knowledge and to change attitudes and behaviors toward more protective actions. We included several factors instead of testing the factors separately, as we were interested in testing the most optimal intervention based on the literature. In both studies, we subsequently measured participants’ attitudes and intentions to take protective actions. In addition to assessing these main variables, we also measured participants’ risk knowledge and experienced fear to check whether the intervention indeed successfully manipulated these variables. In Study 2, we additionally investigated whether the intervention would influence a behavioral decision regarding zoonotic risks. Moreover, we examined whether our designed intervention is more effective than another informative text about zoonoses (which was added as a third condition). In this condition, participants were provided with an informative message by the World Health Organization (WHO) on zoonoses (see Figure 1 for the sequence of events in both studies).

We expected the intervention to change participants’ attitudes, behavioral intentions, and behavioral decisions to take more protective actions compared with the control condition. This was expected as (1) including actions for protective behaviors increases the effectiveness of a message [43,44] and as (2) the PMT factors “probability and severity of the threat”, “self-efficacy”, and “effectiveness of the recommended behavior” contribute to people’s motivation to take protective actions [45,49,51,54]. Furthermore, we explored whether our designed intervention changed participants’ attitudes, behavioral intentions, and behavioral decisions to take more protective actions compared with the WHO information condition.

Both studies were approved by the BMS Ethics Committee of the University of Twente in Enschede, the Netherlands (approval number: Study 1: 220904 and Study 2: 230370). Below, we report the materials and methods and results sections of each study, in which we described all manipulations, measures, and exclusions (if any) in these studies. The materials and data of the reported studies are available via the Open Science Framework (https://osf.io/qchua/, accessed on 9 September 2023).

## 2. Study 1

### 2.1. Materials & Methods

#### 2.1.1. Participants and Design

The sample size was determined a priori. Based on pre-studies, we expected a small to medium effect size (*d* = 0.35). We conducted a sensitivity analysis using G*Power 3.1.9.4 with *d* = 0.35, 95% power, and *α* = 0.05 [55]. According to the “difference between two independent means” statistical test, we needed 178 participants per group (356 total).

In total, 406 (204 female; 199 male; 2 nonbinary/third gender; 1 preferred not to disclose) people participated in this study. Participants’ mean age was 36.05 years (*SD* = 9.81, range = 20–65 years). Participants were recruited via MTurk. They were paid USD 3.00 as a reward.

The design was a between-participants, one-factorial design in which risk communication was manipulated with two levels (intervention vs. control). Participants were randomly assigned to one of the conditions. The dependent variables were intentions to change behavior to prevent future zoonoses and attitudes toward behaviors preventing future zoonoses. Risk knowledge (before and after the manipulation) and fear were measured as manipulation check variables.

#### 2.1.2. Procedure and Materials

This study was conducted on 23 and 24 June 2022. This study was published online via Qualtrics and took about 15 min to complete. Participants read the informed consent and, after agreeing, they started the study. Participants were told that we were interested in investigating people’s experiences, perceptions, and intentions towards zoonoses. The term zoonosis was shortly explained.

First, participants were asked about their gender, age, and whether they have pets (see also Figure 1 above providing an overview of the sequence of events). Then, to measure (pre) risk knowledge, participants were asked to indicate for 10 items regarding risks of zoonoses to what extent they (dis)agreed (7-point scale, 1 = strongly disagree to 7 = strongly agree). These 10 items covered the causes of zoonoses as discussed in the introduction section. Example items are as follows: “Scratches or bites from domestic animals increase the risk for zoonotic infections” and “It is not possible for meat to transmit zoonotic diseases to humans” (reverse-coded).

Then, risk communication was manipulated by either showing participants a message in which information about zoonoses was presented (intervention condition) or by not showing any information (control condition). The intervention message was created using the graphic design platform Canva.com and Figma for the template of the message. Appendix A presents the intervention message. The message described key facts, consequences, and prevention of zoonoses. It included information on the probability and severity of the threat, self-efficacy, and effectiveness of the recommended behavior. The information was presented in succinct writing using bullet points and with the most important information in bold letters. We did not specify the source of the message.

Under key facts, it was explained what zoonoses are, via which animals and how zoonoses are transmitted, and that zoonoses are now more frequent. Under consequences, the severity of zoonoses was emphasized by indicating the possible physical, economic, and social consequences of zoonoses and by indicating that zoonoses are responsible for about one billion cases of illness in people and millions of deaths each year. Furthermore, the possibility of fully occupied hospitals was emphasized to increase fear. Although full hospital beds are not a typical consequence of zoonoses is general, we emphasized this in the message as it is a severe consequence that can occur in a few zoonotic outbreaks. We choose to emphasize this specific severe consequence as previous research showed that people fear hospitals being full the most [56]. We also included a picture from iStock photo showing a gym which was turned into a hospital. The picture displayed a row of full hospital beds and many doctors walking around. Under prevention, it was mentioned to avoid consuming animal products, to avoid animal contact, to wash hands after contact, and, for those who have pets, to regularly visit veterinarians. We did not include specific actions to prevent vector-borne zoonoses, other than having pets checked by their vet. To increase the perceived effectiveness of the recommended behavior, the message included why the proposed behaviors are effective. To increase people’s self-efficacy about the suggested protective actions, we added the phrase “YOU CAN DO IT!” under the mentioned prevention bullet points. Adding this existing self-efficacy measure should increase people’s intentions to take protective actions [29].

To stimulate careful reading, we told participants in the intervention condition that questions would be asked about the message later. Also, they could only proceed after 75 s to prevent participants from easily skipping reading the information. Participants in the control condition did not receive any information message. They were also not provided with any other task and proceeded with the questionnaire.

Then, for all participants, intentions to change their behavior to prevent future zoonoses were measured. We asked them whether they intend to change their behavior concerning the consumption of animal products, contact with domestic and nondomestic animals, and veterinary visits for pets. This was measured using 8 items. Example items are as follows: “With regards to consuming meat I would…” and “With regards to getting into contact with wild animals (e.g., rats, birds, mice, etc.) I would…”. They could indicate whether they intend to show the described behavior (1) more than they currently do, (2) just as much as they currently do, (3) less than they currently do (4) not do it at all, or (5) they could indicate they already did not display this behavior and would stick to that (similar to [14]).

Next, all participants filled in the (post) risk knowledge scale with the same 10 items as in the previously described prerisk knowledge measure. For participants in the intervention condition, this was the second time they filled in this scale, this time after the risk message. For participants in the control condition, this was the first time they filled in the risk knowledge scale.

Then, participants’ attitudes toward protective behavior were measured with 14 items. Example items are “Consuming animal food from intensive livestock farming industries is bad because they increase the risk of me getting zoonoses” and “Getting into contact with wild animals (e.g., rats, birds, mice, etc.) is bad since they could infect me with a zoonotic disease”. Participants responded on a 7-point Likert scale (1 = strongly disagree to 7 = strongly agree).

Afterwards, as a manipulation check, participants’ fear was measured with 6 items. Three items measured experienced fear in general (i.e., “I feel fearful/anxious/frightened”) and three items measured fear for the specific events of hospitals being full, economic crisis, and social disruption (“I fear the hospitals being full”). Participants responded on a 7-point Likert scale (1 = strongly disagree to 7 = strongly agree). At the end of the questionnaire, participants in the intervention condition were asked whether they read the presented information carefully (7-point scale, 1 = strongly disagree, 4 = neither agree, nor disagree, 7 = strongly agree). Four participants indicated to not have read the information carefully (a score equal to or lower than 3). They were omitted from analyses. Finally, participants were thanked for their participation and debriefed.

### 2.2. Results

For all dependent variables, we first assessed whether the assumptions of the analysis of variance (ANOVA) were met. We tested the normality and homogeneity of variances and checked whether outliers distorted the results. For risk knowledge and behavioral intentions, the assumptions on homogeneity of variances were violated and nonparametric tests were performed (Levene tests for risk knowledge: *F*(1, 400) = 5.58, *p* = 0.02, and for behavioral intentions: *F*(1, 400) = 8.26, *p* = 0.004).

#### 2.2.1. Manipulation Checks

Risk Knowledge. After recoding the reversed items, the Cronbach’s alpha of all risk knowledge items was 0.57. Deleting two items (concerning zoos and meat consumption), increased Cronbach’s alpha to 0.73. The results with these two items excluded were similar to the results with all items included. We report the results for the mean of the total scale, as was originally intended.

To test whether the intervention had influenced participants’ risk knowledge, a Mann–Whitney U test was conducted, with risk communication (intervention vs. control) as the independent variable and the mean of all post risk knowledge items as the dependent variable. The analysis showed that participants had a higher post risk knowledge in the intervention condition (*MeanRank* = 221.46, *M* = 4.88, *SD* = 0.70) than in the control condition (*MeanRank* = 181.74, *M* = 4.63, *SD* = 0.55); *U* = 16209.00, *p* = 0.001, *d* = 0.35.

Furthermore, a Wilcoxon signed-rank test within the intervention condition with pre and post risk knowledge test pairs showed that participants in intervention condition had a higher risk knowledge after the risk communication (*M* = 4.88, *SD* = 0.70) than before (*M* = 4.69, *SD* = 0.53), *Z* = −4.16 *p* < 0.001, *η*^2^ = 12.03, (post–pre negative ranks = 61.85; positive ranks = 90.19). See Table 1 for all means and standard deviations.

Fear. A principal component factor analysis showed that all six items loaded on one factor. Cronbach’s alpha was 0.91. An ANOVA with risk communication (intervention vs. control) as the independent variable and fear as the dependent variable was conducted. The analysis showed that participants in the intervention condition experienced more fear (*M* = 4.98, *SD* = 1.32) than participants in the control condition (*M* = 4.58, *SD* = 1.37); *F*(1, 400) = 9.15, *p* = 0.003, *η*_p_^2^ = 0.02, *d* = 0.30. Note that for Study 2, factor analysis indicated two factors: general and specific fear (see Study 2). When analyzing the results according to these two factors, both general and specific fear showed the same results as the analysis of all fear items together, as presented here.

#### 2.2.2. Attitudes toward Protective Behavior

After recoding the reversed items, the Cronbach’s alpha of all items of attitudes was 0.65. Deleting two items (concerning nonorganic consumption and hunting for meat) increased Cronbach’s alpha to 0.77. The results were similar for analyses with all items included and with the two items deleted. We report the results for the mean of the total scale.

To analyze whether the risk communication message had influenced participants’ attitudes toward protective behavior, an ANOVA was conducted with risk communication (intervention vs. control) as the independent variable and attitudes toward protective behavior as the dependent variable. The results showed that participants in the intervention condition had a more positive attitude toward changing their behavior (*M* = 4.77, *SD* = 0.62) than participants in the control condition (*M* = 4.53, *SD* = 0.52); *F*(1, 400) = 17.79, *p* = 0.001, *η*_p_^2^ = 0.04, *d* = 0.42. See Table 2 for all means and standard deviations.

#### 2.2.3. Intentions to Change Protective Behavior

The Cronbach’s alpha of all items of behavioral intentions was 0.86. To analyze whether people intend to change their behavior to reduce zoonotic risks as a result of the risk communication message, a Mann–Whitney U test was conducted with risk communication (intervention vs. control) as the independent variable and the mean of all behavioral intention items (4-point scale: more, equal, fewer, not at all) as the dependent variable. In the analysis, we excluded the scores that indicated that participants already did not show a specific behavior. The results showed that participants in the intervention condition intended to change their behavior more strongly (*MeanRank* = 213.37; *M* = 3.00, *SD* = 0.84) than participants in the control condition (*MeanRank* = 188.75; *M* = 2.85, *SD* = 0.72); *U* = 17826.50, *p* = 0.04, *d* = 0.20 (see also Table 2).

#### 2.2.4. Correlations between Risk Knowledge, Attitudes, Behavior, and Fear

A Pearson correlation was reported for relationships between normal variables. Nonparametric Spearman’s rho was reported for relationships between variables for which rank tests were conducted (risk knowledge and behavioral intentions). Attitudes toward protective actions were relatively weakly, but significantly, positively correlated with behavioral intentions, *r_s_* = 0.12, *n* = 402, *p* = 0.02; relatively strongly correlated with post risk knowledge, *r_s_* = 0.64, *n* = 402, *p* < 0.001; and moderately correlated with fear, *r* = 0.39, *n* = 402, *p* < 0.001. So, the more positive participants’ attitude towards protective behaviors, (1) the more they intend to take on protective behaviors, (2) the more knowledgeable they are about the risks, and (3) the more fear they experience.

Furthermore, behavioral intentions were not related with post risk knowledge, *r_s_* = 0.02, *n* = 402, *p* = 0.69, or with fear, *r_s_* = 0.08, *n* = 402, *p* = 0.11. See also Table 3. As attitudes, knowledge, and fear were all dependent variables, no conclusions can be drawn about their causal relationships. It is possible that, for instance, more fear led participants to be more eager to acquire knowledge, but also that more knowledge led to more fear.

## 3. Study 2

The results of Study 1 showed that informing participants with our designed risk information message successfully changed their attitudes and behaviors to reduce zoonotic risks. As we compared our designed intervention to a no-intervention control condition, we did not know whether our intervention would affect attitudes and behavioral intentions regarding zoonotic risks more strongly compared to another informative text on zoonoses. Therefore, in Study 2, we investigated whether our designed intervention was more successful than another informative text about zoonoses. We choose to compare our intervention with a World Health Organization (WHO) informative text on zoonoses, as the WHO has a directive and coordinating role in the world regarding health.

In Study 1, the intervention message increased participants’ post risk knowledge as compared with the control condition and as compared with the pre risk knowledge measure. Measuring risk knowledge twice in the intervention condition, however, could have biased participants’ responses. On the one hand, participants in the intervention condition had more time to (unconsciously) think about these questions. On the other hand, participants may have wanted to be consistent in their answers, which could explain the relatively small differences between the intervention and control conditions. Therefore, risk knowledge was only measured once (after the manipulation) in Study 2.

As it was recommended to investigate whether the intervention would affect a behavioral decision, we added a behavioral decision regarding zoonotic risks in Study 2. Study 2 also investigated whether the results of Study 1 would be replicated.

### 3.1. Materials & Methods

#### 3.1.1. Participants and Design

The sample size was a priori determined. As in Study 1, we expected a small to medium effect size (*d* = 0.35, *f* = 0.175). We conducted a sensitivity analysis using G*Power 3.1.9.7 with *f* = 0.175, 95% power, and *α* = 0.05 [55]. For the ANOVA statistical test with 3 groups, we needed a total sample size of 510.

In total, 706 (364 female; 333 male; 8 nonbinary/third gender; 1 preferred not to disclose) people participated in this study. Participants’ mean age was 37.99 years (*SD* = 13.00, *range* = 18–81 years). Participants were recruited via Prolific. They were paid GBP 3.00 as a reward.

The design was a between-participants, one-factorial design in which risk communication was manipulated with three levels (intervention vs. WHO vs. control). Participants were randomly assigned to one of the conditions. The dependent variables were attitudes toward behaviors preventing future zoonoses, intentions to change behavior to prevent future zoonoses, and a behavioral decision. Again, risk knowledge and fear were manipulation check variables.

#### 3.1.2. Procedure and Materials

This study was conducted on 30 May 2023. The procedure of Study 2 was similar to the procedure of Study 1, with a few exceptions. First, we added a condition which provided an existing informative message on zoonoses to compare our intervention message with. We used the informative text and picture on zoonoses as published on the WHO website (https://www.who.int/news-room/fact-sheets/detail/zoonoses, accessed on 25 April 2023). We showed this text with a picture in the same outlook as the intervention message. So, neither the WHO website URL nor their logo were included. As for the intervention message, the source of the message was not mentioned.

A second change was that we added a behavioral response measure. Participants were informed that five experience gift vouchers would be raffled among participants. At the end of the questionnaire, they were asked to choose which experience gift voucher they would like to receive if they won a voucher. They could choose between three options with zoonotic risks (e.g., dairy farm experience) and three options without zoonotic risks (e.g., outdoor activity park). A final change was that we measured risk knowledge only once, after the manipulation of the risk communication.

So, participants received either our intervention message, the WHO message, or no message. Then, we measured behavioral intentions (8 items), post risk perception (10 items), attitudes toward self-protective behavior (14 items), fear (6 items), and a behavioral decision on an experience gift voucher (see also Figure 1). In the intervention and WHO conditions, none of the participants indicated not to have read the information carefully (a score equal to or lower than 3).

### 3.2. Results

For all dependent variables, we first assessed whether the assumptions of ANOVA were met by testing the normality and homogeneity of variances and checking for outliers. Two extreme outliers were omitted from analyses as they distorted the results. The homogeinity assumption was violated for attitudes and nonparametric tests were performed (Levene test: *F*(2, 703) = 6.40, *p* = 0.002). See Table 4 for all means and standard deviations.

#### 3.2.1. Manipulation Checks

*Risk Knowledge.* After recoding the reversed items, the Cronbach’s alpha of all items of risk knowledge was 0.86. A one-way ANOVA was performed to compare the effect of risk communication (intervention vs. WHO vs. control) on risk knowledge. This analysis showed an effect of risk communication; *F*(2, 703) = 63.36, *p* < 0.001, *η*_p_^2^ = 0.15, *d* = 0.84. Post hoc tests showed that participants in the intervention condition had a higher risk knowledge (*M* = 5.69, *SD* = 0.76) than both participants in the WHO condition (*M* = 5.55, *SD* = 0.71), *p* = 0.048, *d* = 0.19, and participants in the control condition (*M* = 4.96, *SD* = 0.76), *p* < 0.001, *d* = 0.95. The WHO and control conditions also differed significantly; *p* < 0.001, *d* = 0.80.

*Fear.* A principal component factor analysis showed that all six items loaded on two factors. One factor represented fear experienced in general (i.e., “I feel fearful/anxious/frightened). The second factor represented specific fear (i.e., fear of hospitals being full, fear of an economic crisis, and fear of a social disruption). The Cronbach’s alpha of the general fear items was 0.92. The Cronbach’s alpha of the specific fear items was 0.80. A one-way ANOVA comparing the effect of risk communication (intervention vs. WHO vs. control) on general fear showed that participants did not differ between the risk communication conditions; *F*(2, 703) = 0.52, *p* = 0.60, *η*_p_^2^ = 0.001, *d* = 0.06 (intervention condition: *M* = 2.98, *SD* = 1.53; WHO condition: *M* = 2.84, *SD* = 1.50; control condition: *M* = 2.89, *SD* = 1.50).

A one-way ANOVA comparing the effect of risk communication (intervention vs. WHO vs. control) on specific fears showed a marginally significant effect of risk communication; *F*(2, 703) = 2.48, *p* = 0.08, *η*_p_^2^ = 0.007, *d* = 0.17. Post hoc analyses showed that participants in the intervention condition experienced more specific fear (*M* = 4.41, *SD* = 1.44) than participants in the control condition (*M* = 4.13, *SD* = 1.41), *p* < 0.03, *d* = 0.20. The WHO condition (*M* = 4.21, *SD* = 1.44) did not differ from the intervention, *p* = 0.12, *d* = 0.14, or control condition, *p* = 0.56, *d* = 0.05.

#### 3.2.2. Attitudes toward Protective Behavior

After recoding the reversed items, the Cronbach’s alpha of all items of attitudes was 0.87. To analyze whether the risk communication message influenced participants’ attitudes toward protective behavior, a Kruskal–Wallis test was conducted, with risk communication (intervention vs. WHO vs. control) as the independent variable and attitudes toward protective behavior as the dependent variable. The analysis showed a significant effect of risk communication; *H*(2) = 78.44, *p* < 0.001, *η*_p_^2^ = 0.11, *d* = 0.70. Participants in the intervention condition had a more positive attitude toward changing their behavior (*MeanRank* = 426.59; *M* = 5.22, *SD* = 0.82) than both participants in the WHO condition (*MeanRank* = 371.76; *M* = 5.01, *SD* = 0.74), *p* = 0.002 and participants in the control condition (*MeanRank* = 263.23; *M* = 4.61, *SD* = 0.67), *p* < 0.001. The WHO and control conditions also differed significantly; *p* < 0.001.

#### 3.2.3. Intentions to Change Protective Behavior

The Cronbach’s alpha of all items of behavioral intentions was 0.80. Again, the scores of participants who indicated that they already performed a specific behavior were not included in their means. A one-way ANOVA comparing the effect of risk communication (intervention vs. WHO vs. control) on behavioral intentions showed an effect of risk communication; *F*(2, 703) = 6.58, *p* = 0.001, *η*_p_^2^ = 0.018, *d* = 0.27. Post hoc tests showed that participants in the intervention condition intended to change their behavior more strongly (*M* = 3.09, *SD* = 0.66) than participants in the control condition (*M* = 2.87, *SD* = 0.63); *p* < 0.001, *d* = 0.33. Also, participants in the WHO condition had stronger intentions (*M* = 3.00, *SD* = 0.62) than control participants; *p* = 0.037, *d* = 0.20. The intervention condition did not differ in terms of intentions compared with the WHO condition; *p* = 0.13, *d* = 0.14.

#### 3.2.4. Behavioral Decision

A chi-squared test was conducted, with risk communication (intervention vs. WHO vs. control) as the independent variable and the behavioral decision on the experience gift voucher (with animals vs. without animals) as the dependent variable. The analysis showed a significant effect of risk communication on the behavioral decision; *Χ*^2^(2) = 8.03, *p* = 0.018, *η*^2^ = 0.01, *d* = 0.21. More participants in the intervention condition chose an experience without animals (81.47%; 189 out of 232) compared with the WHO condition (70.94%; 166 out of 234), *Χ*^2^(1) = 7.11, *p* = 0.008, *η*^2^ = 0.01, *d* = 0.21, and compared with the control condition (72.46%; 171 out of 236), *Χ*^2^(1) = 5.35, *p* = 0.02, *η*^2^ = 0.02, *d* = 0.25. The WHO and control conditions did not differ significantly; *Χ*^2^(1) = 0.13, *p* = 0.72, *η*^2^ = 0.0003, *d* = 0.03.

#### 3.2.5. Correlations

A Pearson correlation was reported for relationships between normal variables. Nonparametric Spearman’s rho was reported for relationships between variables for which rank tests were conducted (attitudes and behavioral decision). As in Study 1, attitudes toward protective actions were significantly positively correlated with behavioral intentions, *r_s_* = 0.35, *n* = 706, *p* < 0.001; with post risk knowledge, *r_s_* = 0.66, *n* = 706, *p* < 0.001; with general fear, *r_s_* = 0.25, *n* = 706, *p* < 0.001; and with specific fear, *r_s_* = 0.31, *n* = 706, *p* = 0.002. Also, attitudes were weakly positively related to the behavioral decision, *r_s_* = 0.12, *n* = 702, *p* = 0.001.

This time, behavioral intentions were weakly, but positively correlated with post risk knowledge, *r* = 0.24, *n* = 706, *p* < 0.001; with general fear, *r* = 0.23, *n* = 706, *p* < 0.001; and with specific fear, *r* = 0.16, *n* = 706, *p* < 0.001. Also, behavioral intentions were weakly positively related to the behavioral decision, *r_s_* = 0.16, *n* = 702, *p* < 0.001.

Furthermore, the behavioral decision was weakly, but significantly positively correlated with post risk knowledge, *r_s_* = 0.09, *n* = 702, *p* < 0.001; but not with general fear, *r_s_* = 0.007, *n* = 702, *p* = 0.20, or specific fear, *r_s_* = 0.03, *n* = 702, *p* = 0.043. See also Table 5.

## 4. General Discussion

The results of Studies 1 and 2 showed that the risk communication intervention increased risk knowledge and led to more positive attitudes and behavioral intentions to reduce zoonotic risks compared with the no-intervention control condition. Moreover, the intervention message was more effective than the WHO information message.

The intervention message increased participants’ knowledge of zoonotic risks in both studies. Whereas the effect was small to medium in Study 1, it was large in the more strongly powered Study 2. Although the presented knowledge was succinct, it still influenced the detailed measure of risk knowledge. This suggests that it is not necessary to share a vast amount of knowledge about zoonoses for risk perception to increase.

The intervention also resulted in more positive attitudes towards protective action in both studies. This effect was medium to large. This is important as attitudes play a vital role in the prevention, control, and acceptance of measures [57]. In addition to citizens taking protective actions, governments play an essential role in taking measures to reduce future zoonoses. These measures could include, for instance, reducing the density of livestock farms and facilitating a transition to nonanimal products (see [15,58]).

The risk communication intervention also led to stronger behavioral intentions and decisions to reduce zoonotic risks. This effect was small to medium. Nevertheless, these effect sizes can have important consequences [59]. When interpreting the means for behavioral intentions in the intervention conditions, these reflect a score of participants intending to show protective behaviors more than they currently do (*M*_study 1_ = 3.00; *M*_Study 2_ = 3.09). For the behavioral decision, the increase in the percentage of intervention participants who chose options of reduced zoonotic risks as compared with the already high percentage of WHO and control participants who chose these options is about 10% (81.47 vs., respectively, 70.94% and 72.46%).

Correlational analyses showed that behavioral intentions were related to attitudes towards protective actions, but not, or very weakly, to risk knowledge and to fear. This suggests that for the information message to be effective on a behavioral level, it should focus more on the actions people could take rather than on increasing their risk perception (likelihood and severity of threat). Meta-analyses showed that all PMT factors contribute to people’s motivation to take protective actions [45,49,51,54]. Nevertheless, the findings of six meta-analytic studies showed that it is more important to present information aimed at increasing the effectiveness of the recommended behavior and self-efficacy than to present information aimed at increasing risk perceptions and fear to motivate people to take action [51]. Our results are in line with these findings.

Furthermore, correlational analyses showed that the behavioral decision was only weakly, but significantly, related to behavioral intentions. First, meta-analyses suggest that intentions explain about 20% of the variance in behavior [60,61,62]. In addition to the intention–behavior gap, this weak relationship can furthermore be explained by the fact that the behavioral decision focused on one aspect of the behaviors that were covered by the behavioral intentions. In addition to preventing contact with animals and their environments, the behavioral intentions also focused on animal product intake, hygiene, and vet visits. Future research could, for instance, investigate whether the intervention message would affect participants’ consumption behavior.

Our designed intervention was more effective than the WHO information message on zoonoses regarding risk knowledge, attitudes, and the behavioral decision. The results showed that the WHO message did increase risk knowledge and positive attitudes towards protective actions compared with the control condition, but not as strongly as our intervention. Furthermore, the WHO message was as effective as the intervention message in changing people’s behavioral intentions to reduce zoonotic risks, but did not affect the behavioral decision. A combination of multiple aspects on which the intervention and WHO message differ could have caused the differences in effectiveness. First, post hoc analyses showed that the intervention message increased experiences of specific fear, whereas the WHO message did not. This difference in specific fear may have been due to the intervention message emphasizing the probability and severity of the threat, which the WHO message did not emphasize. This may have influenced participants’ feelings of urgency to process the information in the message and to be motivated to change their actual behavior (e.g., [51,52]). Furthermore, the intervention message focused entirely on what citizens can do to prevent future zoonoses, whereas the WHO message did not direct their message specifically to citizens. The WHO message also included information on what the agricultural sector and authorities should do and which developments the WHO is working on to reduce zoonotic risks. This may have influenced participants’ feelings of responsibility to change their behaviors. Moreover, the WHO message did not focus on presenting information about the effectiveness of the recommended behavior, nor on people’s self-efficacy about the recommended behavior, whereas the intervention message did. These factors are important motivators of behavioral change (e.g., [49,51]). Finally, the WHO message was presented using a more advanced, less understandable language level, and a longer, less succinct text. Consequently, the WHO message might have been harder for participants to process, leading to a less strongly increased risk knowledge as compared with our intervention condition. Importantly, for the intervention message as well as the WHO message, the source of the message was not specified. So, the differences between these conditions cannot be explained by possible differences in the credibility of explicitly mentioned sources. It is possible, however, that some participants assumed that the messages had been created by the academics running the study.

As the effectiveness of risk messages depends on many factors, including the credibility of the source and the audience (e.g., [52,63], we need to be careful generalizing the findings about the effectiveness of our message to all circumstances, especially as fear appeal messages can be rejected or can even be counterproductive (e.g., [51]). Messages that are attributed to an expert source are generally more effective, but not always very strongly [52,64,65,66]. The effect of source credibility on the effectiveness of a message also depends on the specific message, the channel used, and the receivers [52]. Research conducted during the COVID-19 pandemic showed that willingness to take the suggested actions (e.g., getting vaccinated) was more strongly related to trust in scientists than to trust in the government [67]. This research additionally showed that there was less compliance with the suggested actions people could take in countries with low trust in scientists. Future research should investigate the effectiveness of our intervention message when presented by various sources (e.g., academics, national governments, and international institutions [e.g., the WHO]) and in countries with differing levels of trust.

Whereas the generalizability of our findings when the message is presented by other sources needs to be investigated, we have no reason to believe the findings cannot be generalized to a broader population. In our studies, MTurk and Prolific workers conducted the studies voluntarily. It is possible that people who volunteered to take part in our studies differed from people who do not volunteer [68]. For instance, people are more likely to participate if they are interested in the topic [69]. This tendency for more interested people to participate is reduced when monetary incentives are offered [69], which was the case in our studies. Furthermore, our studies had a low drop-out rate (Study 1 = 2.38%; Study 2 = 3.27%), had a diverse sample regarding age and gender, and were sufficiently powered. Also, note that participants who volunteered were randomly assigned to one of the conditions, which makes it highly likely that the participant groups are comparable [70]. So, we have no reason to believe the results are not generalizable to a broader population. It is conceivable, though, that a small group of people were either not at all or negatively affected by the intervention due to resistance. Resistance occurs when a message is too far removed from the receiver’s position [71]. As people who could possibly show resistance feel involved in the topic as well, we do not expect them to have been underrepresented in these studies.

The results of our research add to previous research on zoonotic risk communication messages (e.g., [40,41,42]) by focusing on zoonoses in general instead of a specific zoonotic disease (such as Ebola). With one basic and short risk communication message focusing on all zoonoses together, we were able to efficiently affect participants’ attitudes and behavioral intentions to reduce risks of all zoonoses instead of only one zoonotic disease. A message focusing on a specific zoonotic disease may be more effective in changing people’s behavior toward that specific disease, but a general message may reduce the risk of a variety of zoonoses. Furthermore, our research extends previous research on zoonotic risk communication eby including recommendations for protective actions people could take. This is important for the effectiveness of the message as people often do not know which actions are effective (e.g., [39]) and do not take action otherwise (e.g., [43,44]). With our intervention, we are the first to experimentally demonstrate that a general zoonotic risk communication message changes attitudes and behavioral intentions to take more protective actions to reduce zoonotic risks. These results also provide an important insight for policy measures on how to communicate zoonotic risks to the general public to reduce zoonotic outbreaks of a broad range of diseases.

## 5. Conclusions

To conclude, in the present research we designed a literature-based intervention providing risk knowledge about zoonoses, which actions people could take to reduce zoonotic outbreaks, and a fear appeal. The intervention message included information about the probability and severity of the risk, self-efficacy, and the effectiveness of the recommended behavior. This intervention changed participants’ attitudes, behavioral intentions, and their behavioral decision to take more actions to reduce zoonotic risks and was more effective than the WHO information message on zoonoses. The results imply that a basic and short risk information message based on scientific literature has the potential to reduce zoonotic risks.

## Figures and Tables

**Table 1:** Means and standard deviations for risk knowledge (pre and post) per condition of Study 1 (higher scores mean a higher perception of zoonotic risk).

Risk Communication	Pre Risk Knowledge		Post Risk Knowledge	
	*M*	*SD*	*M*	*SD*
Yes	4.69 _a_	0.53	4.88 _b_	0.70
No	4.63 _a_		0.55	

Note 1: Means with different subscripts (a, b) differ significantly from each other (*p* < 0.05). Note 2: Risk perception in the control condition was measured once.

**Table 2:** Means and standard deviations for behavior change, attitudes toward protective behavior, and fear in Study 1 (higher scores mean, respectively, more intentions to display protective behavior, more positive attitudes toward protective behavior, more risk knowledge, and more fear).

Risk Communication	Intentions to Change Behavior		Attitudes toward Protective Behavior		Fear	
	*M*	*SD*	*M*	*SD*	*M*	*SD*
Intervention	3.00 _a_	0.84	4.77 _a_	0.62	4.98 _a_	1.32
Control	2.85 _b_	0.72	4.53 _b_	0.52	4.58 _b_	1.37

Note: Means with different subscripts (a, b) differ significantly from each other within a column (*p* < 0.05).

**Table 3:** Correlations between attitude, behavioral intentions, and fear in Study 1 (N = 402). For attitude, behavioral intentions, and behavioral decisions, a higher score means reducing zoonotic risks. For fear, a higher score indicates more fear.

	Attitude	Behavioral Intentions	Post Risk Knowledge
Behavioral intentions	*r_s_* = 0.12, *p* = 0.017		
Post risk knowledge	*r_s_* = 0.64, *p* < 0.001	*r_s_* = 0.02, *p* = 0.69	
General fear	*r* = 0.39, *p* < 0.001	*r_s_* = 0.08, *p* = 0.11	*r_s_* = 0.26, *p* = 0.002

Note: Pearson correlation was reported for relationships between normal variables. Nonparametric Spearman’s rho was reported for relationships between variables for which rank tests were conducted (risk knowledge and behavioral intentions).

**Table 4:** Means and standard deviations for intentions to change behavior, attitudes toward protective behavior, risk knowledge, and fear in Study 2 (higher scores mean, respectively, more intentions to display protective behavior, more positive attitudes toward protective behavior, more risk knowledge, and more fear).

Risk Communication	Intentions to Change Behavior		Attitudes toward Protective Behavior		Post Risk Knowledge		General Fear		Specific Fear	
	*M*	*SD*	*M*	*SD*	*M*	*SD*	*M*	*SD*	*M*	*SD*
Intervention	3.09 _a_	0.66	5.22 _a_	0.82	5.69 _a_	0.76	2.98 _a_	1.53	4.41 _a_	1.44
WHO	3.00 _a_	0.62	5.01 _b_	0.74	5.55 _b_	0.71	2.84 _a_	1.50	4.21 _a,b_	1.44
Control	2.87 _b_	0.63	4.61 _c_	0.67	4.96 _c_	0.76	2.89 _a_	1.50	4.13 _b_	1.41

Note 1: Means with different subscripts (a, b, c) differ significantly from each other within a column (*p* < 0.05).

**Table 5:** Correlations between attitude, behavioral intentions, behavioral decision, and fear in Study 2 (N = 706, except for behavioral decision, where N = 702). For attitudes, behavioral intentions, and behavioral decision, a higher score means reducing zoonotic risks. For fear, a higher score indicates more fear.

	Attitude	Behavioral Intentions	Behavioral Decision	Post risk knowledge	General Fear
Behavioral intentions	*r_s_* = 0.35 (*p* < 0.001)				
Behavioral decision	*r_s_* = 0.12 (*p* = 0.001)	*r_s_* = 0.16 (*p* < 0.001)			
Post risk knowledge	*r_s_* = 0.66 (*p* < 0.001)	*r* = 0.24 (*p* < 0.001)	*r_s_* = 0.09 (*p* = 0.024)		
General fear	*r_s_* = 0.25 (*p* < 0.001)	*r* = 0.23 (*p* < 0.001)	*r_s_* = 0.05 (*p* = 0.20)	*r* = 0.11 (*p* = 0.002)	
Specific fear	*r_s_* = 0.31 (*p* < 0.001)	*r* = 0.17 (*p* < 0.001)	*r_s_* = 0.03 (*p* = 0.43)	*r* = 0.16 (*p* < 0.001)	*r* = 0.54 (*p* < 0.001)

Note: Pearson correlation was reported for relationships between normal variables. Nonparametric Spearman’s rho was reported for relationships between variables for which rank tests were conducted (attitudes and behavioral decision).

**Figure A1:**
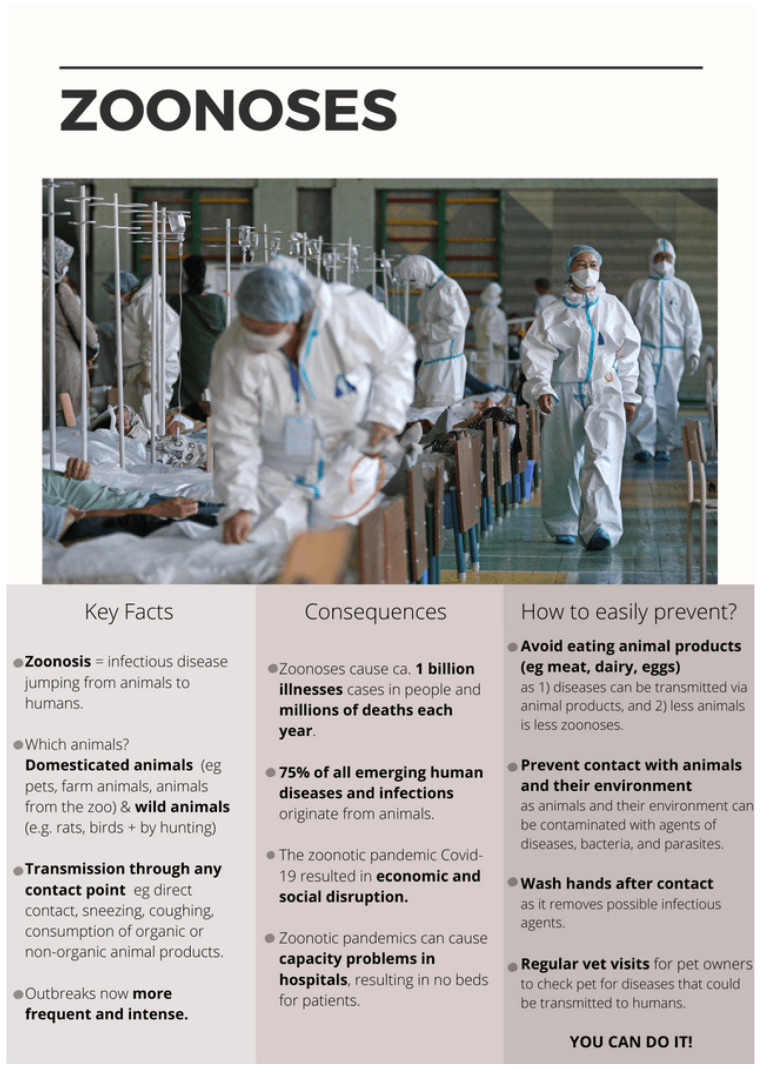
Intervention message of Studies 1 and 2.

## Data Availability

The materials and data of the reported studies are available via the Open Science Framework: https://osf.io/qchua/ (accessed on 9 September 2023).
